# Personality Traits and Identity Styles in Methamphetamine-Dependent Women: A Comparative Study

**DOI:** 10.5539/gjhs.v8n1p14

**Published:** 2015-05-15

**Authors:** Seyed Kaveh Hojjat, Ebrahim Golmakani, Mohammad Hossein Bayazi, Razieh Mortazavi, Mina Norozi Khalili, Arash Akaberi

**Affiliations:** 1 Addiction and Behavioral Sciences Research Center, North Khorasan University of Medical Sciences, Bojnurd, Iran; 2 Department of Anesthesiology, Faculty of Medicine, Mashhad University of Medical Sciences, Mashhad, Iran and Addiction and Behavioral Sciences Research Center, North Khorasan University of Medical Sciences, Bojnurd, Iran; 3 Department of Psychology, Torbate Jam Branch, Islamic Azad University, Torbate Jam, Iran; 4 Islamic Azad University, Torbate Jam Branch, Torbate Jam, Iran; 5 Department of Community Medicine, School of Medicine, North Khorasan University of Medical Sciences. Bojnurd, Iran; 6 Department of Epidemiology and Biostatistics, Sabzevar University of Medical Sciences, Sabzevar, Iran; 7 School of Continuing Studies, McGill University, Montreal, QC, Canada

**Keywords:** identity styles, methamphetamine, personality traits, adult female respondents

## Abstract

**Background:**

Studies over the past two decades have shown that various personality traits of substance-dependent men measure differently than compared to normal individuals. However fewer studies have addressed the role of identity as an influential factor in the onset and continuation of drug dependency.

**Methods:**

The objective of this study was to compare the Big Five personality factors and identity styles in methamphetamine dependent women and non-user group. Forty eight methamphetamine dependent women under treatment in Welfare Organization’s residential centers filled out the NEO Five-Factor Inventory (NEO-FFI) and the Berzonsky’s Identity Style Inventory. They were compared with 48 non-dependent women who were matched in terms of age, education, marital status, and occupation. Data was analyzed with t student test. Statistical analyses were performed using the SPSS V.16 software. Differences were considered significant at P<0.05.

**Results:**

Results found that methamphetamine dependent woman had significantly higher levels of neuroticism and lower levels of conscientiousness, agreeableness and openness to experience compared to normative sample of female respondents. In addition, mean scores of diffuse/avoidant identity style in methamphetamine user women was significantly higher than non-user group. This is while non-user women had a significantly higher mean in normative identity style.

**Conclusion:**

Identity styles along with personality traits can be a key role in drug use in women in this study. Therefore, enhancing understanding about the role of identity can be helpful in treatment programs especially in harm reduction approaches.

## 1. Introduction

Drug abuse is considered one of the most entrenched and life altering health related difficulties in today’s world (Drugs & Crime, 2003). The United Nations Office for Drug Control (UNODC) listed addiction as one of the four crises in the world. This office reported that the prevalence of substance abuse is constantly increasing (Drugs & Crime, 2003). Most people think that drug addiction is a masculine phenomenon, while drug abuse in women has remained hidden for various reasons such as social stigma about women’s addiction and the centrality of women in the family (Cooper, Agocha, & Sheldon, 2000). Various scientific findings indicate that substance abuse in women yields a higher risk of a variety of health problems than substance abuse in men (Kay, Taylor, Barthwell, Wichelecki, & Leopold, 2010). In recent years, the prevalence of drug abuse has increased in both men and women. Based on research reports, the pace of increase among women is significantly higher than men. In most countries, addiction treatment and harm reduction programs tailored to the needs of addicted women either don’t exist or are very rare. Therefore, lack of attention to gender-related needs leads to increased vulnerability in addicted women (Rahimimovaghar, Nejatisafa, Mohammadi, & Izadian, 2011).

Substance dependency is one of the major health problems in Iran and it can lead to psychological, physical and social problems (Ziaaddini, Sharifi, Nakhaee, & Ziaaddini, 2010). Studies show that Iran has the highest rate of opium use in the world (Mehrjerdi et al., 2013; Rahimimovaghar et al., 2011). Co-use of heroin with methamphetamine (MA) is a new epidemic health concern among Iranian female drug users.

### 1.1 Personality Traits and Drug Use

Personality is made up of a combination of distinguishing characteristics called traits. Traits refer to a distinctive set of attributes such as thinking, feeling, attitude, and behavior (McCrae & Terracciano, 2005). Costa and McCrae define personality relying on the Big Five factors model and/which includes neuroticism, extraversion, openness to experience, agreeableness, and conscientiousness. Neuroticism is the tendency to experience anxiety, stress, hostility, impulsivity, shyness, irrational thought, depression and low self-esteem. Extraversion is the tendency to be positive, assertive, dynamic, kind, and sociable. Openness to experience is the tendency to be curious, interested in arts, intellectual, flexible, creative, and innovative. Agreeableness is the tendency to be forgiving, kind, generous, trusting, sympathetic, obedient, devoted, and loyal. And finally, conscientiousness is the tendency to be organized, efficient, dependable, restrained, logic oriented, and reflective (De Fruyt, De Bolle, McCrae, Terracciano, & Costa, 2009). Several studies have also successfully established a positive correlation between personality traits and the use of certain substances (Anderson, Tapert, Moadab, Crowley, & Brown, 2007; Grekin, Sher, & Wood, 2006; Kornor & Nordvik, 2007; Prisciandaro, McRae-Clark, Moran-Santa Maria, Hartwell, & Brady, 2011; Terracciano, Lockenhoff, Crum, Bienvenu, & Costa, 2008). Individuals with high Neuroticism with negative emotions and low Agreeableness, and those who are undisciplined and disorganized (low Conscientiousness) are more likely to use substance than those who have opposite of these traits (Sutin, Evans, & Zonderman, 2013).

One of the various realities in the course of human growth and development, which is considered an important developmental task, is the search for self-conception or acquiring an identity; the feeling that “Who am I?”, “What social status do I have?”, and “What do I want from life?” (Tsang, Hui, & Law, 2012). Erikson hypothesized that identity is the conscious sense of self which is made up of values, beliefs, and goals. According to Erikson, the function of identity is to create harmony between an individual’s self-image as a unique person and the perception of others about him. In other words, Erikson believed that identity is the establishment of balance between oneself and others (Tsang et al., 2012). Most research in the field of identity carried out in past decades was based on the Marcia model. But in a newer contemporary model, Berzonsky (2005) emphasizes on those social-cognitive processes that cause individuals in the society to choose different identity styles (Berzonsky & Kuk, 2005). Berzonsky believes that individuals are placed in different conditions based on their data processing method and adapt one of the three identity processing styles called diffuse-avoidant, normative, and informational (Berzonsky & Kuk, 2005). In individuals with a diffuse avoidant identity, data processing in carried out based on the individual’s emotions and feelings. Individuals with normative processing style follow the actions and behaviors of the majority of people (Berzonsky & Ferrari, 2009). They usually show little flexibility in dealing with life difficulties. Individuals with informational processing style make decisions based on reasoning and thought in any situation. They have a flexible approach in dealing with various life issues (Berzonsky & Ferrari, 2009; Berzonsky & Kuk, 2005). Some studies reported that diffuse-avoidance identity style was related with higher delinquent behaviors and substance and alcohol abuse in adolescents (Adams, Munro, Munro, Doherty-Poirer, & Edwards, 2005; Good, Grand, Newby-Clark, & Adams, 2008).

In the present study, the personality traits and identity styles of methamphetamine dependent women were compared with those of non-using women, in order to acquire a better understanding of this dark part of the personality. Women who use stimulant drugs are in danger of displaying other risky behaviors due to the effect of this type of substance on their psychological condition and behavior. We hypothesized that identity styles are different in MA user women and relation between identity and certain personality traits can be a moderator for use of substance. Therefore, knowing more about the relation of identity and personality profile can be helpful in treatment programs especially in harm reduction approaches in MA user women. Aim of the study to compare the identity style beside the personality factors in methamphetamine dependents women and non user group to found better understanding about position of identity in etiology of addiction.

## 2. Methods

### 2.1 Participants

Ninety six female respondents were enrolled in the study, of which 48 were MA dependent and 48 non-users (the control group). Patients under study were chosen from women admitted in Welfare Organization’s addiction rehabilitation centers. These women referred from their family physicians or social workers in north khorasan province for detoxification and rehabilitation of methamphetamine dependency. Participants admitted in two residential drug rehabilitation center in bojnurd and shirvan city in north khorasan province, northeastern of Iran.

Inclusion criteria for substance-dependent women includes: Diagnosis of methamphetamine dependency according to DSM IV TR, lapse of at least two weeks from residence in drug rehabilitation center, absence of methamphetamine psychosis based on a psychiatric interview, consent for taking part in the study, and having at least an elementary level of education.

The control group was made up of individuals introduced by participating patients. Patients were asked to introduce someone from their relatives, friends, or an acquaintance that were similar to themselves in terms of age, marital status, education, being employed or unemployed and economic status, but were not using any substance. Subjects matched one by one. Next, the researcher contacted the introduced person and explained the study’s nature. In case of consent, the researcher approached the respondent. After carrying out a routine urine test for drug dependency and obtaining a negative result, the individual filled out the questionnaires.

### 2.2 Measures

#### 2.2.1 The NEO-Five Factory Inventory Was Used to Assess Personality Traits

Costa and McCrae (2008) reported an alpha coefficient of 0.74 to 0.89 for the Big Five Personality Inventory with a mean of 0.81 (Costa & McCrae, 2008). The internal consistency of this questionnaire ranges from 0.68 to 0.86 and the test retest reliability after two weeks was 0.86 to 0.90 for the five measures (Robert R McCrae & Costa, 2004). In the Persian version the reliability coefficients obtained for the five factors was: neuroticism=0.83, extraversion=0.80, openness to experience=0.80, agreeableness=0.79 and conscientiousness=0.79 (Grosifreshi, Mehryar, &Ghazitabatabaei, 2001).

#### 2.2.2 Berzonsky’s Identity Style Inventory Was Used to Assess Identity Styles

It includes 40 items: 11 items for informational style, 9 items for normative style, 10 items for diffuse-avoidant style, and 10 items for commitment. Items are scored on a scale ranging from 1 (not at all like me) to 5 (very much like me) (White, Wampler, & Winn, 1998). Regarding this inventory, Crosby et al. (2009) reported a Cronbach’s alpha of 0.64 for informational style, 0.58 for normative style, 0.75 for diffuse-avoidant style and 0.72 for commitment (Crocetti, Rubini, Berzonsky, & Meeus, 2009). The Persian version was tested by Aghajani, who reported a reliability of 0.73 for informational style, 0.69 for diffuse avoidant style, and 0.68 for commitment (Aghajani, 2002).

### 2.3 Statistical Analysis

The statistical methods used in this study were the Independent T-test for comparing two means and Chi-squared tests for comparing qualitative variables in the two groups. We used MANOVA for compare the NEO and Berzonsky subscales. In this study SPSS 16 software was used for data analysis and the significance level was 0.05.

### 2.4 Ethical Considerations

This research approved by Regional University of medical sciences Ethics committee. All participants provided written consent stating their willingness to participate in this study.

## 3. Results

The mean age of the substance dependent group was 30.33±8.38 and non-user group was 31.39±8.94. Based on statistical test results, the two groups showed no significant difference in terms of age (p=0.546) and educational level (p=0.328). The educational status of the respective samples is presented in Table 1.

**Table 1:** Educational status of samples in two groups. Values are numbers (percentages)

Group	Middle school or lower	Diploma	Associates	Bachelors or higher	Total
MA users	10(20.8)	25(52.1)	8(16.7)	5(10.4)	48(100)
Non-users	8(16.3)	20(42.9)	8(16.3)	12(24.5)	48(100)
Total	18(18.6)	45(47.4)	16(16.5)	17(17.5)	96(100)

The mean scores of NEO big five personality factors in the two groups are presented in Table 2. According to MANOVA analysis there was a significant difference between the two groups. (ΛWilks=0.769, p<0.001)

**Table 2:** Comparison of NEO dimension scores in MA user women and non-user group

Personality factors	Groups	mean ± SD	Mean difference	Significance
**Neuroticism**	**MA user**	7.82± 39.32	5.94	0.001
	**Non-user**	7.82 ±33.38		
**Extraversion**	**MA user**	5.96 ±38.08	-2.28	0.053
	**Non-user**	4.86± 40.36		
**Openness to experience**	**MA user**	3.76± 35.16	-3.78	0.001
	**Non-user**	4.52±38.94		
**Agreeableness**	**MA user**	6.17± 37.86	-5.78	0.001
	**Non-user**	5.41±43.64		
**Conscientiousness**	**MA user**	9.25±40.57	-5.27	0.004
	**Non-user**	6.08±45.84		

The MA user women’s group showed higher levels of neuroticism compared to the non-abuser group. Openness to experience, agreeableness, and conscientiousness was significantly higher in the non-user group.

The mean scores of the Berzonsky’s Identity Styles in the two groups are shown in Table 3. According to MANOVA analysis, there was a significant difference between the two groups in Berzonsky’s Identity Styles (λWilks=0.694, p <.001).

**Table 3:** Comparison of mean scores of identity styles in MA abuser women and non-abuser group

Identity Styles	groups	mean ± SD	Mean Difference	Significance
**Informational orientation**	**MA user**	6.66±37.49	1.76	0.194
	**Non-user**	5.88±39.25		
**Normative orientation**	**MA user**	29.87 ±29.87	3.04	0.014
	**Non-user**	4.87±32.92		
**Diffuse/avoidant orientation**	**MA user**	5.71±32.51	-6.12	0
	**Non-user**	6.27±26.4		
**Commitment**	**MA user**	6.69 ± 32	5.25	0
	**Non-user**	5.77±37.25		

Results from Berzonsky’s Identity Style Inventory also showed a significant difference in the normative, diffuse/avoidant, and commitment orientations (Table 3). Mean scores of MA user women in the diffuse/avoidant and commitment orientation was significantly higher than non-user women, while the non-user women had a significantly higher mean in normative orientations.

## 4. Discussion

In this study, we compared the Big Five personality factors and identity styles in MA abuser women and non-user group. Results obtained indicate that out of the Big Five personality factors, mean neuroticism in MA user women is significantly higher than in non user group. However, MA user women obtained significantly lower scores in the dimensions of openness to experience, conscientiousness, and agreeableness compared to non-user women. Also the results concerning the identity styles showed that scores of MA user women in the diffuse/avoidant orientation was significantly higher than non-user respondents. Furthermore, the non-user women also had a significantly higher mean in normative orientations.

Our finding concerning the positive correlation between the traits high Neuroticism, Low Agreeableness and low Conscientiousness in MA user are consistent with the results of Anderson et al., 2007; Grekin et al., 2006; Kornor & Nordvik, 2007; Prisciandaro et al., 2011; Sutin et al., 2013; Terracciano et al., 2008). However, in these studies, type of substance was not considered as a variable. Neuroticism refers to the tendency to experience negative emotions such as fear, sadness, impulsivity, and vulnerability to pressure (De Fruyt et al., 2009). These personality characteristics somehow justify the individual’s resort to unconventional methods such as drug use in the shape of self medication (Loukas, Krull, Chassin, & Carle, 2000). Lack of acceptable levels of conscientiousness in these individuals doubles the necessity of applying methods to put emphasis on the “consequences of addiction”(although a very logical inference it is clear that many of these hypothesis have not proven effective- although from difference schools of thinking and focusing on alcoholism and other drugs).

Our results showed that MA user had no significant differences in Extraversion as compared to non user group. Our finding was consistent with results of Satin et al (2013) and Terraacciano et al. (2008) and inconsistent with results of Carter et al study (Norwegian study) (Carter et al., 2001; Sutin et al., 2013; Terracciano et al., 2008). Results in Norwegians study showed high Neuroticism, low Conscientiousness, low Extraversion and Low Agreeableness in opium users. In a Meta analysis entitled “The relationship between the five factor of personality and symptoms of psychiatric disorder”, Malouff indicated that there are a variety of clinical disorders directly associated with high Neuroticism, low Conscientiousness, Low Agreeableness and low Extraversion. Low extraversion is associated with symptoms of anxiety, mood disorders, and psychotic disorders (Malouff, Thorsteinsson, & Schutte, 2005). In this research we did not study the psychotic disorders resulting from the use of MA and no significant difference in extraversion in this group may be related to this issue. Although there does not seem to be supporting evidence for this hypothesis.

Our results showed that the diffuse/avoidant identity style was significantly higher in the user group compared to non-user group. These results were consistent with results of research carried out by Nurmi et al. (1997), Jones et al. (1992), White et al. (1998). All of these researchers found a significant difference in the drug addicts’ choice of the diffuse avoidant style (Jones, Ross, & Hartmann, 1992; Nurmi, Berzonsky, Tammi, & Kinney, 1997; White et al., 1998). White’s study also displayed that this identity style is associated with repeated relapses of addiction and difficulties in recovering from substance abuse (White, Montgomery, Wampler, & Fischer, 2003). The case group in our research rely on a type of substance in itself indicative of severe involvement. The latter remains relatively consistent with White and Fisher’s findings about higher relapse rates and difficulties in treatment. Individuals with a diffuse/avoidant orientation show less attention to the long-term consequences of their actions. Their momentary emotions and feelings play a key role in their decision making. Such individuals postpone dealing with their problems as far as possible and their behavior seems driven by immediate outcomes (Berzonsky & Ferrari, 2009). They have a tendency to rely on short-term measures rather than long-term reforms Berzonsky & Ferrari, 2009). The use of drugs with the incentive to eliminate unpleasant feelings is the most obvious sign of this type of orientation in individuals with drug addiction.

## 5. Conclusion

It can be said that addicted individuals in this research have obvious differences in their personality traits and identity styles compared to the non user group. This is consistent with other studies carried out in this field. Considerable differences between the drug user group and the non user group in some of their personality traits and choice of identity styles suggests further research in this field. It seems necessary to assess other variables such as irrational beliefs, defense mechanisms, and maladaptive schemas in addicted and normal individuals besides assessing their identity style and we suggest to other researcher to compare identity styles beside of these variable in drug users. This way we can find the missing pieces to the puzzle of addictive behaviors in this group of patients. It should be noted that this study had substantial limitations. One of the most important limitations was the small sample size which was related to low access to MA user women and their lack of cooperation for taking part in the study. In addition, the admittance of these patients in addiction rehabilitation residential centers might have affected their responses to the questionnaires, compared to the control group who were not in such conditions. This issue was of course inevitable.
